# Supplementary figures and images for: Bleb analysis using anterior segment optical coherence tomography after trabeculectomy with amniotic membrane transplantation
Source: PLoS One. 2023 May 3;18(5):e0285127. doi: 10.1371/journal.pone.0285127 (PMC10155995; doi:10.1371/journal.pone.0285127)

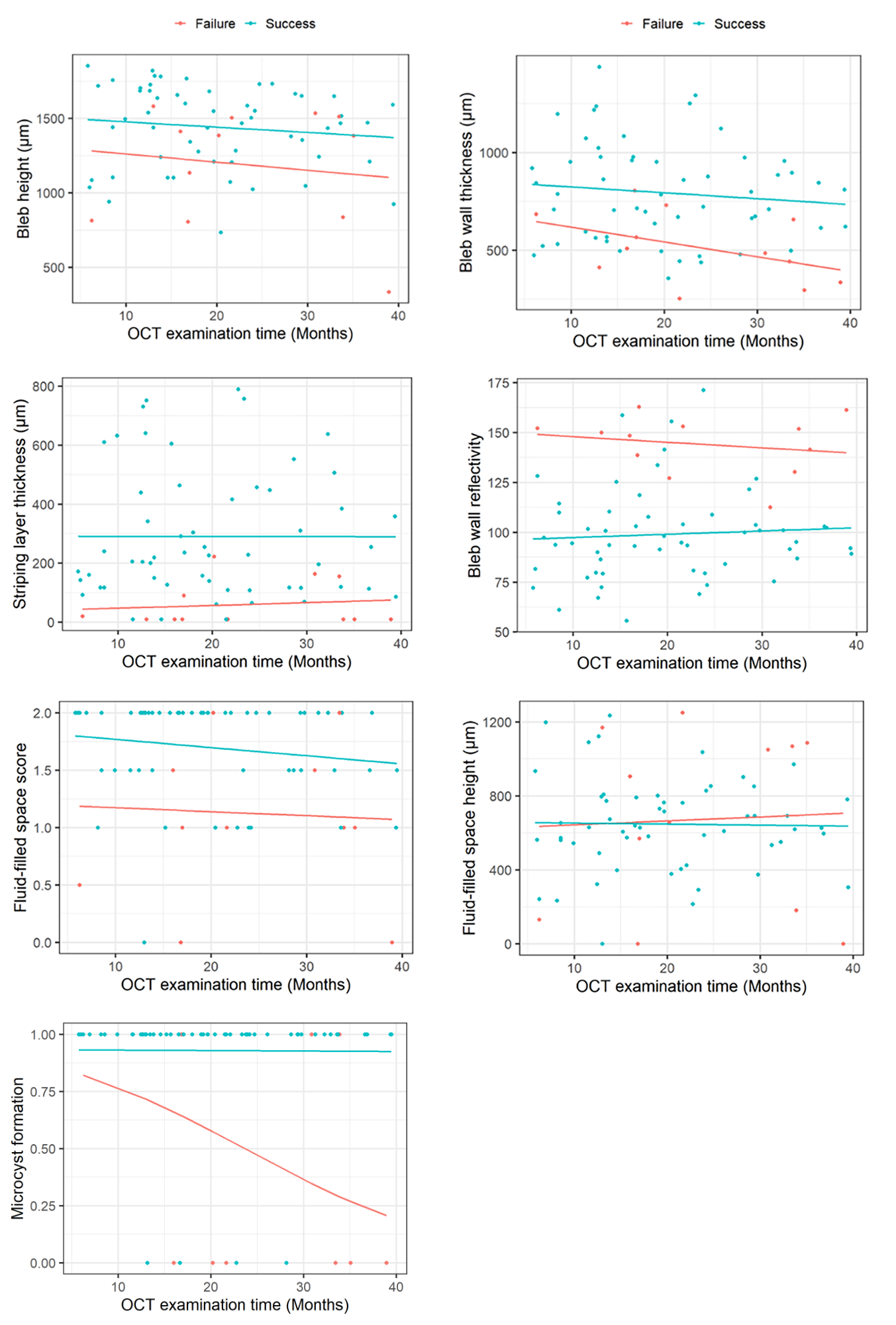

Supplement: S1 Fig — There were no significant interaction effects between the AS-OCT examination time and groups in all the bleb parameters (P ≥ 0.333 for all). (TIF) [file pone.0285127.s001.tif]
