# Supplementary material for: Bleb analysis using anterior segment optical coherence tomography after trabeculectomy with amniotic membrane transplantation
Source: PLoS One. 2023 May 3;18(5):e0285127. doi: 10.1371/journal.pone.0285127 (PMC10155995; doi:10.1371/journal.pone.0285127)
Supplement: Attachment — Submitted filename: PONE-D-22-33214-Comments.docx [file pone.0285127.s002.docx]

The manuscript ID: PONE-D-22-33214; entitled “Bleb analysis using anterior segment optical coherence tomography after trabeculectomy with amniotic membrane transplantation” is an interesting and great peer article in the ocular research field The article highlights the potential of AS-OCT imaging techniques as a tool for bleb analysis after surgery. The article covers the basic and translational fundamentals of imaging techniques with enough patient data. The author must be revised the comments for a better understanding of the readers in some of the sections as details mentioned below:

**Comment 1:** In the abstract section, add one line about “why you are doing this work and the importance of the work” and then come to the objective of the study.

**Comment 2:** In the introduction section, page number 3, lines 44-45 add the other fibrosis regulators like epigenetic modification (SAHA), etc. contribution, and limitation. See the reference below:

Ref. “Sharma et al 2016. Epigenetic Modification Prevents Excessive Wound Healing and Scar Formation After Glaucoma Filtration Surgery. Invest Ophthalmol Vis Sci. 2016 Jun 1;57(7):3381-9. doi: 10.1167/iovs.15-18750. PMID: 27367506; PMCID: PMC4961058”.

**Comment 3:** In the introduction section, page number 4, line 77 has to restructured due to this is not the first study. See the reference below:

Ref. “Kim H, Moon S, Kim J, Lee J. The Effect of Amniotic Membrane Transplantation on Trabeculectomy in Patients with Pseudoexfoliation Glaucoma. J Ophthalmol. 2022 Jul 30;2022:9355206. doi: 10.1155/2022/9355206. PMID: 35942064; PMCID: PMC9356778.”.

**Comment 4:** In the result section, page number 14, lines 248-250, define the figure 4 legend each box plots to A-G, for better clarity for the readers.

**Comment 5:** In the result section, page number 15, lines 265-290, the figure number not matched the figure (Figure 4 should be Figure 5). I think, the authors are explaining figure 5, and due to typos or accidental errors, they have mentioned figure 4 in every place rather than figure 5.

**Comment 6:** In the discussion section, page number 16, line 293 has to restructured due to this is not the first study. See the reference below:

Ref. “Kim H, Moon S, Kim J, Lee J. The Effect of Amniotic Membrane Transplantation on Trabeculectomy in Patients with Pseudoexfoliation Glaucoma. J Ophthalmol. 2022 Jul 30;2022:9355206. doi: 10.1155/2022/9355206. PMID: 35942064; PMCID: PMC9356778.”.

In summary, the review article ID: ID: PONE-D-22-33214; entitled “Bleb analysis using anterior segment optical coherence tomography after trabeculectomy with amniotic membrane transplantation” is good and worth publication in the PlosOne journal after addressing these minor comments.
