# Supplementary material for: Bleb analysis using anterior segment optical coherence tomography after trabeculectomy with amniotic membrane transplantation
Source: PLoS One. 2023 May 3;18(5):e0285127. doi: 10.1371/journal.pone.0285127 (PMC10155995; doi:10.1371/journal.pone.0285127)
Supplement: Attachment — Submitted filename: Response to Reviewers.docx [file pone.0285127.s003.docx]

Dear, Prof. Rajiv R. Mohan.

Academic Editor

PLOS ONE

Attached please find the revised manuscript entitled “*Bleb analysis using anterior segment optical coherence tomography after trabeculectomy with amniotic membrane transplantation*”. A point-by-point response to each of reviewer’s comments follows. The authors wish to thank the Editorial Board for their thoughtful consideration and recommendations, and hope that this revised manuscript now meets your requirements for publication.

**Reviewer #1**: The manuscript ID: PONE-D-22-33214; entitled “Bleb analysis using anterior segment optical coherence tomography after trabeculectomy with amniotic membrane transplantation” is an interesting and great peer article in the ocular research field. The article highlights the potential of AS-OCT imaging techniques as a tool for bleb analysis after surgery. The article covers the basic and translational fundamentals of imaging techniques with enough patient data. The author must be revised the comments for a better understanding of the readers in some of the sections as details mentioned below:

**Comment 1**: In the abstract section, add one line about “why you are doing this work and the importance of the work” and then come to the objective of the study.

**Response**: We added the following background of this study in the abstract section.

“Little has been known about the intrableb structures associated with bleb function after trabeculectomy with amniotic membrane transplantation (AMT).”

**Comment 2**: In the introduction section, page number 3, lines 44-45 add the other fibrosis regulators like epigenetic modification (SAHA), etc. contribution, and limitation. See the reference below:
Ref. “Sharma et al 2016. Epigenetic Modification Prevents Excessive Wound Healing and Scar Formation After Glaucoma Filtration Surgery. Invest Ophthalmol Vis Sci. 2016 Jun 1;57(7):3381-9. doi: 10.1167/iovs.15-18750. PMID: 27367506; PMCID: PMC4961058”.

**Response**: We added the other fibrosis regulators in the introduction section as belows:

“An experimental study reported that suberoylanilide hydroxamic acid, a histone deacetylase inhibitor prevented excessive wound healing and scar formation in a rabbit model of glaucoma filtration surgery.[9]” (Invest Ophthalmol Vis Sci. 2016;57:3381–3389.)

**Comment 3**: In the introduction section, page number 4, line 77 has to restructured due to this is not the first study. See the reference below:
Ref. “Kim H, Moon S, Kim J, Lee J. The Effect of Amniotic Membrane Transplantation on Trabeculectomy in Patients with Pseudoexfoliation Glaucoma. J Ophthalmol. 2022 Jul 30;2022:9355206. doi: 10.1155/2022/9355206. PMID: 35942064; PMCID: PMC9356778.”.

**Response**: The bleb analysis with anterior segment optical coherence tomography was not assessed in the previous study of our group (J Ophthalmol. 2022 Jul 30;2022:9355206.). We mentioned the previous study in the introduction section along with another study from our study group.

**Comment 4**: In the result section, page number 14, lines 248-250, define the figure 4 legend each box plots to A-G, for better clarity for the readers.

**Response**: We added the following explanation for each Figure 4A to G.

“(A) Bleb was higher in the success group than that in the failure group. (B) Bleb wall was thicker in the success group than that in the failure group. (C) Stripping layer was thicker in the success groups than that in the failure group. (D) Bleb wall reflectivity was lower in the success group than that in the failure group. (E) Fluid-filled space score was greater than that in the failure group. (F) There was no significant difference in the fluid-filled space height between the two groups. (G) Microcyst formation was more frequently noted in the success group than in the failure group.”

**Comment 5**: In the result section, page number 15, lines 265-290, the figure number not matched the figure (Figure 4 should be Figure 5). I think, the authors are explaining figure 5, and due to typos or accidental errors, they have mentioned figure 4 in every place rather than figure 5.

**Response**: We corrected the figure number from 4 to 5.

**Comment 6**: In the discussion section, page number 16, line 293 has to restructured due to this is not the first study. See the reference below:
Ref. “Kim H, Moon S, Kim J, Lee J. The Effect of Amniotic Membrane Transplantation on Trabeculectomy in Patients with Pseudoexfoliation Glaucoma. J Ophthalmol. 2022 Jul 30;2022:9355206. doi: 10.1155/2022/9355206. PMID: 35942064; PMCID: PMC9356778.”.
**Response**: The bleb analysis with anterior segment optical coherence tomography was not assessed in the previous study of our group (J Ophthalmol. 2022 Jul 30;2022:9355206.). We mentioned the previous study in the introduction section along with another study from our study group.

**Reviewer #2**: This research was about anterior segment optical coherence tomographic findings after trabeculectomy with amniotic membrane transplantation to find out bleb characteristics by using mainly anterior segment OCT and slit-lamp and authors finally searched surgical success predictor.
The topic is very interesting and useful for readers who are Glaucoma surgeon and read PLOSONE. Moreover as authors described in this manuscript already, wound healing and wound modulation is quite important in management of glaucoma.
However, there are a few studies regarding AS-OCT after trabeculectomy with AMT. Therefore, this manuscript look meaningful study because it provides clinically significant important information with AS-OCT.
However, the study has some mild concerns and requires minor revisions and clarification in order to meet the journal's requirements.

**Comment 1**. As described by the authors, the timing of anterior segment OCT is quite different. Please provide the range of the timing of anterior segment OCT in Table 1 (If possible, please provide the routine exam timing in usual clinical settings.)
**Response**: The interval between surgery and AS-OCT examination ranged from 6 to 39 months (median, 19 months). We added the range of anterior segment OCT examination time in Table 1.

**Comment 2**. Please provide the reason that surgical success was defined as IOP < 18 mmHg?

**Response**: We used a stringent criterion for success of trabeculectomy with amniotic membrane transplantation in this study. Previous studies suggested that IOP < 21 mmHg may not be optimal success criteria for trabeculectomy (Ophthalmology 2006;113:930 –936) (J Glaucoma 2017;26:303–310). Recent clinical trials have adopted cut off points of IOP < 18 mmHg based on long-term outcomes of glaucoma surgery (Am J Ophthalmol. 2000 Oct;130(4):429-40.) (Ophthalmology. 2001 Nov;108(11):1943-53.).

We added the above reason why surgical success was defined as IOP < 18 mmHg in the discussion section.

“We used a stringent criterion for success of trabeculectomy with AMT in this study. Previous studies suggested that IOP < 21 mmHg may not be optimal success criteria for trabeculectomy.[40,41] Recent clinical trials have adopted cut off points of IOP < 18 mmHg based on long-term outcomes of glaucoma surgery.[42,43]”

**Comment 3**. And if possible, please describe or discuss the clinical postoperative changes of anterior segment OCT. (In usual, the bleb height might be lower or other morphological changes can be occurred during long-term follow-up)
**Response**: There are a few studies which analyzed longitudinal changes in intrableb structure using AS-OCT after glaucoma surgery (Br J Ophthalmol 2018;102:796–801.) (J Glaucoma. 2021;30:988-995.). Narita et al. compared AS-OCT parameters between 2 weeks and 1 year after trabeculectomy and found that bleb height, bleb wall thickness and ratio of hyporeflective space of the bleb wall tended to decrease in the unsuccessful IOP control group while bleb height and ratio of hyporeflective space of the bleb wall significantly increased in the successful IOP control group at 1 year post-trabeculectomy (Br J Ophthalmol 2018;102:796–801.). With regard to qualitative AS-OCT parameter, the appearance ratios of cyst-like structures and striping phenomenon decreased in both groups (Br J Ophthalmol 2018;102:796–801.). Dangda et al. also reported that reduction in microcysts were noted at intermediate (6 to 12 months) and long-term (>12 months) timepoints compared to early postoperative period (≤ 3 months) after Sub-Tenon's implantation of the Xen Gel stent (J Glaucoma. 2021;30:988-995.). In this study, however, longitudinal changes in the intrableb structure after trabeculectomy with AMT was not assessed. Therefore, further research is needed in order to evaluate longitudinal changes in the intrableb structures with AS-OCT after trabeculectomy with AMT.”

We added following sentences in the limitation section.

“Third, longitudinal changes in the intrableb structure after trabeculectomy with AMT was not assessed in this study. Narita et al.[20] compared AS-OCT parameters between 2 weeks and 1 year after trabeculectomy and found that bleb height, bleb wall thickness and ratio of hyporeflective space of the bleb wall tended to decrease in the unsuccessful IOP control group while bleb height and ratio of hyporeflective space of the bleb wall significantly increased in the successful IOP control group at 1 year post-trabeculectomy. With regard to qualitative AS-OCT parameter, the appearance ratios of cyst-like structures and striping phenomenon decreased in both groups. Dangda et al.[44] also reported that reduction in microcysts were noted at intermediate (6 to 12 months) and long-term (>12 months) timepoints compared to early postoperative period (≤ 3 months) after sub-Tenon's implantation of the Xen Gel stent. Further research is needed in order to evaluate longitudinal changes in the intrableb structures with AS-OCT after trabeculectomy with AMT.”

Sincerely yours,

Jiwoong Lee, MD, PhD.

Department of Ophthalmology, Pusan National University College of Medicine

#179 Gudeok-ro, Seo-gu, Busan 49241, Korea

Tel: +82-51-240-7958

Fax: +82-51-242-7341

Email: alertlee@naver.com
