# Supplementary material for: Bleb analysis using anterior segment optical coherence tomography after trabeculectomy with amniotic membrane transplantation
Source: PLoS One. 2023 May 3;18(5):e0285127. doi: 10.1371/journal.pone.0285127 (PMC10155995; doi:10.1371/journal.pone.0285127)
Supplement: Attachment — Submitted filename: Response to Reviewers.docx [file pone.0285127.s004.docx]

Dear, Nader Hussien Lotfy Bayoumi

Academic Editor

PLOS ONE

Attached please find the revised manuscript entitled “*Bleb analysis using anterior segment optical coherence tomography after trabeculectomy with amniotic membrane transplantation*”. A point-by-point response to the comments follows. The authors wish to thank the Editorial Board for their thoughtful consideration and recommendations, and hope that this revised manuscript now meets your requirements for publication.

**Comment 1**: Please add to the results section, the duration between cataract surgery and the Trab surgery in the eyes that were pseudophakic, if this information was available and study the effect of this duration on the success-failure of the procedure and bleb appearance.

**Response**: We investigated the duration between cataract extraction surgery and the trabeculectomy with AMT and evaluated the effect of this duration on the surgical outcome and intrableb parameters. We added the following sentences in the Result section.

“The effect of duration between cataract extraction and trabeculectomy with AMT on surgical failure and relationship between the duration and intrableb structures were evaluated. The duration between surgeries ranged from 6 to 321 months and the average was 76 ± 73 months. The duration was not associated with surgical failure in univariate logistic regression analysis (odds ratio = 0.999, P = 0.800). All intrableb parameters were not related to the duration (P ≥ 0.297 for all) except for microcyst formation (r = 0.353, P = 0.035).”

We also added following sentences in the Discussion section.

“In the subgroup analysis for pseudophakic eyes, the duration between cataract extraction and trabeculectomy with AMT was not significantly associated with surgical failure. In the correlation analysis, the duration was positively correlated to microcyst formation. Damage of blood aqueous barrier after cataract extraction surgery is likely to persist for a long period of time.[40] (Eye, 2000, 14.1: 61-63.) However, it is also possible to recover over time, suggesting that a positive correlation between the duration and microcyst formation.[41] (British Journal of Ophthalmology. 1991;75: 106–110.)"

**Comment 2**: Please move the part of the literature review (in the limitations of the study) to the body of the discussion section, before the limitations of the study, and restrict the limitations to just mentioning the fact that "Third, longitudinal changes in the intrableb structure after trabeculectomy with AMT was not assessed in this study."

**Response**: We moved the part of the literature review before the limitation section shown as below.

“Narita et al.[20] compared AS-OCT parameters between 2 weeks and 1 year after trabeculectomy and found that bleb height, bleb wall thickness and ratio of hyporeflective space of the bleb wall tended to decrease in the unsuccessful IOP control group while bleb height and ratio of hyporeflective space of the bleb wall significantly increased in the successful IOP control group at 1 year post-trabeculectomy. With regard to qualitative AS-OCT parameter, the appearance ratios of cyst-like structures and striping phenomenon decreased in both groups. Dangda et al.[46] also reported that reduction in microcysts were noted at intermediate (6 to 12 months) and long-term (>12 months) timepoints compared to early postoperative period (≤ 3 months) after sub-Tenon's implantation of the Xen Gel stent. As longitudinal changes in the intrableb structure after trabeculectomy with AMT was not assessed in this study, further research is needed in order to evaluate longitudinal changes in the intrableb structures with AS-OCT after trabeculectomy with AMT.”

We restricted the third limitation shown as below.

“Third, longitudinal changes in the intrableb structure after trabeculectomy with AMT was not assessed in this study.”

**Comment 3**: Please add to the limitations of the study that it was not compared to eyes subjected to Trab without AMT, if it was the target of the authors to demonstrate the additive effect of AMT on Trab with MMC for POAG.

**Response**: Although the purpose of this study was not to demonstrate the additive effect of AMT on trabeculectomy with MMC for patients with POAG, we added following sentences in limitation section.

“Fourth, we did not compare the intrableb structures between trabeculectomy with AMT group and without AMT group.”

Sincerely yours,

Jiwoong Lee, MD, PhD.

Department of Ophthalmology, Pusan National University College of Medicine

#179 Gudeok-ro, Seo-gu, Busan 49241, Korea

Tel: +82-51-240-7958

Fax: +82-51-242-7341

Email: alertlee@naver.com
